# CD33/CD3-bispecific T-cell engaging (BiTE®) antibody construct targets monocytic AML myeloid-derived suppressor cells

**DOI:** 10.1186/s40425-018-0432-9

**Published:** 2018-11-05

**Authors:** Regina Jitschin, Domenica Saul, Martina Braun, Sehmus Tohumeken, Simon Völkl, Roman Kischel, Michael Lutteropp, Cedric Dos Santos, Andreas Mackensen, Dimitrios Mougiakakos

**Affiliations:** 1 Department of Internal Medicine 5, Hematology and Oncology, University of Erlangen-Nuremberg, Ulmenweg 18, 91054 Erlangen, Germany; 2 Amgen Research GmbH, Munich, Germany; 3 Clinical Biomarkers and Diagnostics, Amgen Inc., South San Francisco, CA USA

**Keywords:** Acute myeloid leukemia, Myeloid derived suppressor cells, Bispecific antibodies

## Abstract

Acute myeloid leukemia (AML) is the most common acute leukemia amongst adults with a 5-year overall survival lower than 30%. Emerging evidence suggest that immune alterations favor leukemogenesis and/or AML relapse thereby negatively impacting disease outcome. Over the last years myeloid derived suppressor cells (MDSCs) have been gaining momentum in the field of cancer research. MDSCs are a heterogeneous cell population morphologically resembling either monocytes or granulocytes and sharing some key features including myeloid origin, aberrant (immature) phenotype, and immunosuppressive activity. Increasing evidence suggests that accumulating MDSCs are involved in hampering anti-tumor immune responses and immune-based therapies. Here, we demonstrate increased frequencies of CD14^+^ monocytic MDSCs in newly diagnosed AML that co-express CD33 but lack HLA-DR (HLA-DR^lo^). AML-blasts induce HLA-DR^lo^ cells from healthy donor-derived monocytes in vitro that suppress T-cells and express indoleamine-2,3-dioxygenase (IDO). We investigated whether a CD33/CD3-bispecific BiTE® antibody construct (AMG 330) with pre-clinical activity against AML-blasts by redirection of T-cells can eradicate CD33^+^ MDSCs. In fact, T-cells eliminate IDO^+^CD33^+^ MDSCs in the presence of AMG 330. Depletion of total CD14^+^ cells (including MDSCs) in peripheral blood mononuclear cells from AML patients did not enhance AMG 330-triggered T-cell activation and expansion, but boosted AML-blast lysis. This finding was corroborated in experiments showing that adding MDSCs into co-cultures of T- and AML-cells reduced AML-blast killing, while IDO inhibition promotes AMG 330-mediated clearance of AML-blasts. Taken together, our results suggest that AMG 330 may achieve anti-leukemic efficacy not only through T-cell-mediated cytotoxicity against AML-blasts but also against CD33^+^ MDSCs, suggesting that it is worth exploring the predictive role of MDSCs for responsiveness towards an AMG 330-based therapy.

**Electronic supplementary material:**

The online version of this article (10.1186/s40425-018-0432-9) contains supplementary material, which is available to authorized users.

## Main text

Acute myeloid leukemia (AML) is the most common acute leukemia amongst adults. The disease course is typically aggressive and despite therapeutic advances only 30% of the patients will be long-term survivors. Emerging evidence suggests that immune evasion in AML favors relapse and could antagonize novel immunotherapeutic concepts [1].

Over the last years, myeloid derived suppressor cells (MDSCs) have been gaining momentum in cancer research as promoters of tumor immune escape. MDSCs represent a heterogeneous population that morphologically resembles monocytes or granulocytes sharing some features: myeloid origin, immature phenotype, and T-cell suppressive activity. Accumulating MDSCs have been described in AML patients [2], in myelodysplasia (MDS) [3], and in murine AML models [4]. In fact, AML-blasts hold the potential to induce MDSCs (from conventional monocytes) by exosomal transfer of MUC-1 [2]. These cells could contribute to immune escape partly explaining why AML-blasts despite expressing antigens recognizable to host T-cells (e.g. WT1) rarely are eradicated by the host’s immune system [5]. Targeting MDSCs in preclinical cancer models has shown efficacy in delaying disease thus suggesting further clinical exploitation [6].

Bispecific T-cell engaging (BiTE®) antibody constructs simultaneously target tumor antigens of interest and the T-cell receptor complex. T-cells can be recruited in an antigen-independent manner [7]. The first BiTE® developed against CD33, which is expressed on the majority of AML-blasts, is AMG 330 (Amgen, Thousand Oaks, CA). Preclinical studies revealed its capacity to recruit and to expand autologous T-cells leading to AML-blasts lysis [8, 9]. In fact, CD33 might have an advantage over other targets (e.g. CD123) since it is also expressed on monocytic MDSCs [10]. In this study we sought out to investigate whether AMG 330 could simultaneously confer two hits by redirecting T-cells against both CD33^+^ AML-blasts and CD33^+^ MDSCs thereby further enhancing anti-leukemic immune activity.

**Fig. 1:**
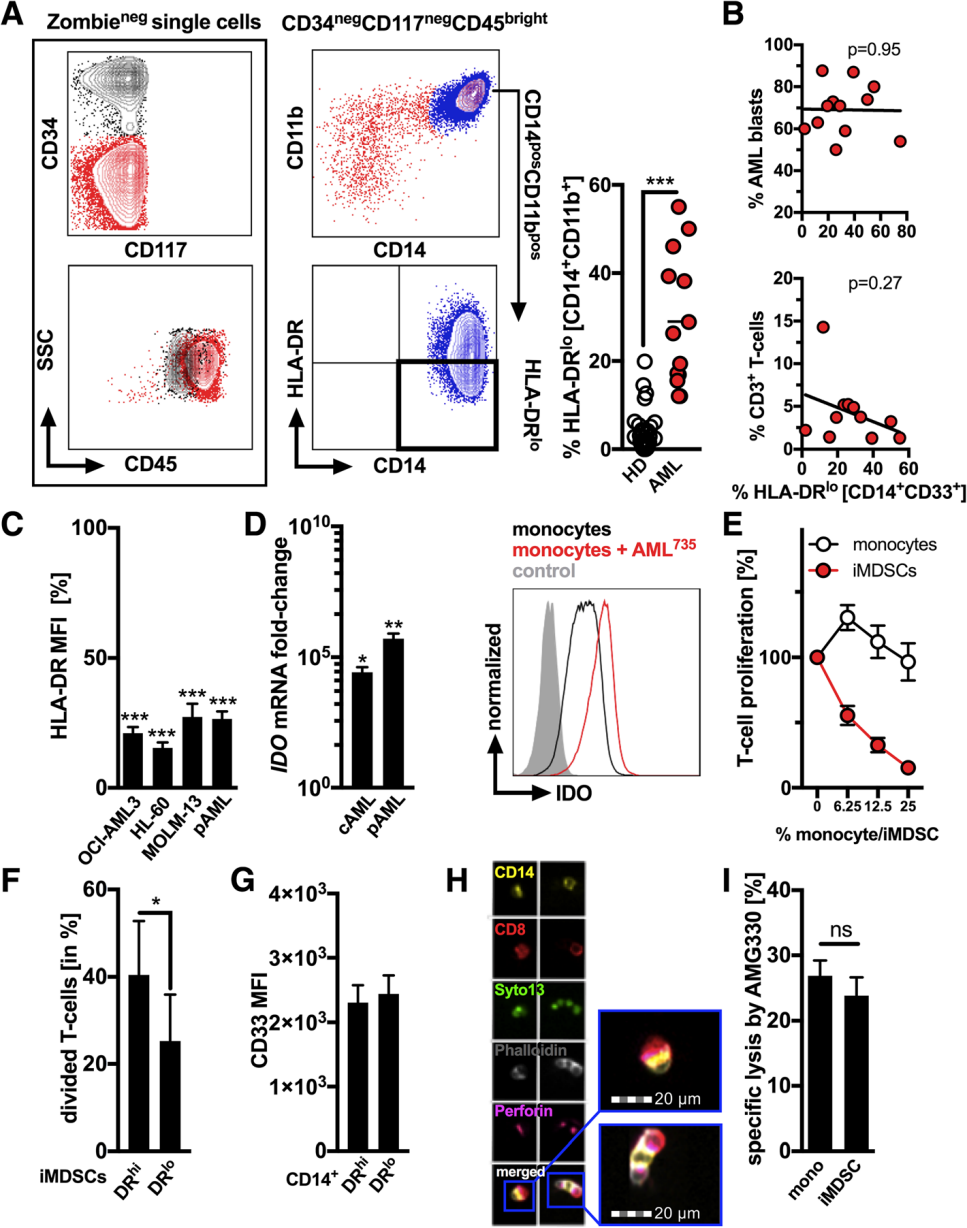
Primary AML-blasts and AML cell lines promote induction of CD14^+^ CD33^+^ IDO^+^ HLA-DR^lo^ MDSCs that are targeted by AMG 330. (**a**) A representative FACS-based analysis of MDSCs within AML patient-derived PBMCs is shown. HLA-DR expression was analyzed in CD14^+^ CD11b^+^ monocytic cells (right panels) among the viable (Zombie^neg^) CD45^hi^ CD34^neg^ CD117^neg^ (left panels) cell fraction. Frequency of HLA-DR^lo^ cells was assessed in untreated AML patients (*n* = 13) and healthy controls (HD, *n* = 37). (**b**) Correlation of the proportion of HLA-DR^lo^ cells within the CD14^+^ population (n = 13) with frequency of circulating AML-blasts (upper panel) and of CD3^+^ T-cells (lower panel). (**c**) Expression levels of HLA-DR based on the median fluorescence index (MFI) were measured by FACS on HD-derived purified CD14^+^ monocytes following 5 days of culture in the presence/absence of AML cell lines (OCI-AML3, HL-60, and MOLM-13, *n* = 5) and primary AML-blasts (*n* = 9). Monocytes cultured alone are set as 100%. (**d**) The relative gene expression (mRNA) of indoleamine-2,3-dioxygenase (IDO) in monocytes cultured in the presence/absence of AML cell lines (cAML, *n* = 6) and primary AML-blasts (pAML, *n* = 10) was semi-quantified by qPCR. Monocytes cultured alone are set as 1. A representative (for *n* = 10) histogram of a FACS analysis of IDO expression in monocytes cultured alone (control) or in presence of primary AML-blasts as shown for cells from the patient with the unique patient number 735 (AML^735^). (**e**) The dose-dependent suppressive activity of HD-derived monocytes and HD-derived monocytes re-educated by cAML (=induced MDSCs (iMDSCs)) was evaluated in co-cultures with VPD450-labeled autologous T-cells activated using anti-CD2, -CD3, and -CD28 microbeads. T-cell proliferation was assessed based on the VPD450 dye dilution after 5 days by FACS and compared with stimulated T-cells alone (set as 100%). (**f**) Suppressive activity of FACS-sorted HLA-DR^hi^ and HLA-DR^lo^ iMDSCs was separately evaluated in co-culture experiments (*n* = 4) with autologous T-cells. (**g**) Cell surface expression of CD33 was semi-quantified by FACS on HLA-DR^lo^ and HLA-DR^hi^ CD14^+^ monocytes (*n* = 10). (**h**) Representative (for at least three independent experiments) FACS**-**imaging of cytotoxic CD8^+^ T-cells (red) conjugated with autologous CD14^+^ monocytes (yellow). Nucleic acids were counter-stained with Syto13 (green), actin with phalloidin (grey), and perforin formation additionally visualized (pink). (**i**) Calcein-labeled monocytes or iMDSCs (*n* = 10) were co-cultured with pre-stimulated autologous T-cells at a 1:10 ratio for three hours and the release of calcein measured using fluorimetric assay. Bars indicate the standard error of the mean. Abbreviations: *, *p* < 0.05; **, *p* < 0.01; ***, *p* < 0.001

First, CD14^+^CD11b^+^CD33^+^ monocytic cells expressing low levels of HLA-DR (HLA-DR^lo^) and resembling one of the most established human MDSC-like phenotype [11] as previously described by us in chronic lymphocytic leukemia (CLL) and malignant melanoma [10, 12] were quantified in the peripheral blood of patients with newly diagnosed AML. A representative flow cytometry (FACS)-based gating strategy is displayed in Fig. 1a, whereby AML-blasts were defined as CD117^+^ and/or CD34^+^ cells during initial AML diagnosis. The proportion of HLA-DR^lo^ cells among monocytes was significantly increased in AML patients as compared to healthy controls (HD) (28.98 ± 4.19%, *n* = 13 versus 3.28 ± 0.75%, *n* = 37) in line with previous observations [2]. In fact, MDSCs can be cytogenetically related to the malignant AML clone as recently reported [2]. Percentage of aberrant monocytes did not correlate (positively) with the frequency of circulating myeloid blasts or (negatively) with the frequency of T-cells in contrast to findings from CLL [10], B-NHL [13], and MDS [3] (Fig. 1b).

CD14^+^ monocytes isolated from HD were co-cultured for three to five days with AML cell-lines (OCI-AML3, HL-60, and MOLM-13/cAML) or primary AML-blasts (pAML) that were previously labeled with a vital dye for better discriminating both populations in ultra-low attachment surface plates allowing full recovery of monocytes. Presence of AML-blasts led (at day five) to a significant reduction of HLA-DR expression in CD14^+^ monocytes (Fig. 1c). Previous studies as for example in CLL and in patients following allogeneic stem cell transplantation have shown that the monocytic MDSCs can express indoleamine-2,3-dioxygenase (IDO) [10, 14]. In fact, IDO-mediated tryptophan depletion and production of kynurenine can modulate T-cell responses. Furthermore, IDO has been shown to negatively impact efficacy of immune-based therapies such as of T-cells carrying chimeric antigen receptors [15] while high kynurenine concentrations predict an unfavorable outcome in AML patients [16]. We detected a significant IDO upregulation on the gene expression and protein level in monocytes upon contact to cAML- and pAML-blasts (Fig. 1d). As anticipated, the functional assessment of AML-educated monocytes co-cultured at different ratios with activated autologous T-cells revealed a strong T-cell suppressive activity (as compared to non-AML-educated monocytes), which is in line with their MDSC-like phenotype (Fig. 1e) and with previous observations in AML [2], allowing us to denominate them as induced MDSCs (iMDSCs). Next, we separated the HLA-DR^hi^ and HLA-DR^lo^ fraction among the bulk of AML-educated monocytes using FACS-based cell sorting and then repeated the T-cell suppression assays. Here, we observed that the HLA-DR^lo^ subset still holds the strongest T-cell suppressive capacity further confirming the enhanced regulatory features of HLA-DR^lo^ monocytic iMDSCs (Fig. 1f).

We further assessed whether T-cells can be engaged by AMG 330 to target autologous monocytes and/or T-cell suppressive (AML-educated) iMDSCs. Noticeably, previous studies have shown that AMG 330-mediated lysis is co-determined by the cell surface CD33 levels [8, 9]. The median fluorescence intensity (MFI) of CD33 as assessed by flow cytometry was comparable in both the HLA-DR^lo^ (=MDSCs) and HLA-DR^hi^ CD14^+^ subsets (Fig. 1g). Purified CD3^+^ T-cells engaged by AMG 330 were able to form immunological synapses with autologous CD14^+^ cells as revealed by the F-actin and perforin polarization (Fig. 1h) and which is an important determinant for the efficacy T-cell based immune therapies [17]. Specific lysis of calcein-labeled HD-derived monocytes triggered by AMG 330 in presence of autologous T-cells was at comparable levels as for their iMDSC counterparts that had been previously educated by AML cell-lines and despite their elevated IDO expression and their T-cell suppressive activity (Fig. 1d-e, i).

**Fig. 2:**
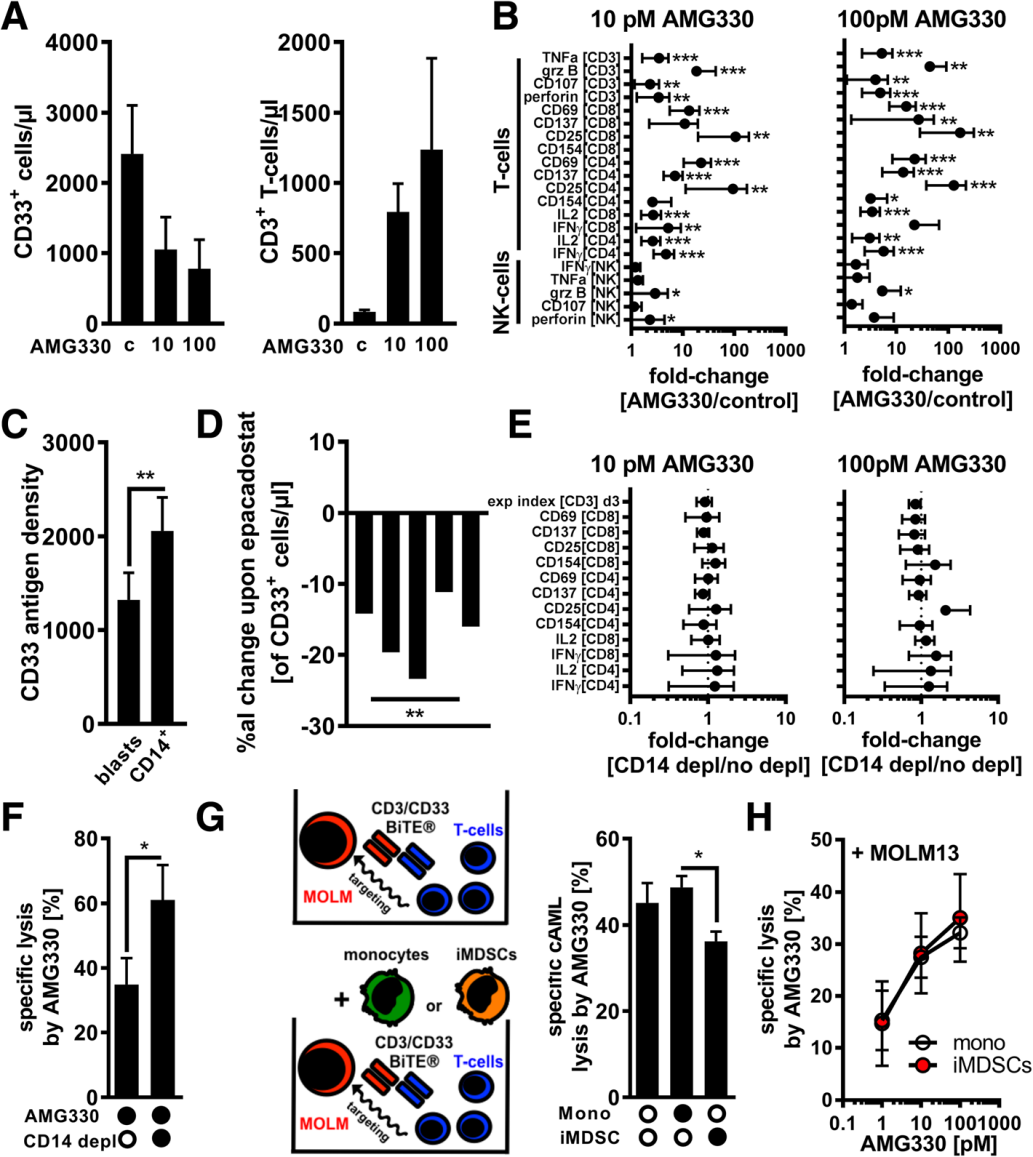
AMG 330 triggers T-cell-mediated lysis of AML-blasts that is further enhanced by MDSC depletion. (**a**) The absolute number of CD33^+^ AML-blasts and CD3^+^ T-cells was quantified in patient-derived AML PBMCs (*n* = 10) after 6 days of treatment with control BiTE® antibodies (**c**) or AMG 330. (**b**) AML-derived PBMCs (*n* = 12) were treated with control BiTE® antibodies or AMG 330 for three days. The median fluorescence intensity (MFI) of TNFα, granzyme B (grz B), CD107, perforin, CD69, CD137, CD25, CD154, IL2, and IFNγ was assessed by FACS in CD4^+^/CD8^+^ CD3^+^ T-cells and CD56^+^CD3^neg^ NK-cells as indicated. The cells’ MFI from samples treated with control antibodies was set as 1. (**c**) CD33 surface antigen quantification was performed for AML-blasts and CD14^+^ monocytes (*n* = 8). (**d**) AML-derived PBMCs (*n* = 5) were treated with 10 pM AMG 330 in the presence or absence of the IDO inhibitor epacadostat (1 μM) and the number of CD33^+^ AML-blasts quantified. The graph displays the individual %al changes in cell numbers in presence of epacadostat. (**e**) AML PBMCs (*n* = 7) with/without prior depletion of CD14^+^ cells were treated with AMG 330 for three days. Expansion index and MFI of CD69, CD137, CD25, CD154, IL2, and IFNγ were assessed by FACS in VPD450-labeled CD4^+^/CD8^+^ CD3^+^ T-cells. Samples without depletion of CD14^+^ cells were set as 1. (**f**) AML-derived PBMCs (n = 5) with/without prior depletion of CD14^+^ cells were treated with AMG 330 for six days. LDH release as a surrogate for cell lysis was measured in the cultures’ supernatants. (**g**) Calcein-labeled MOLM-13 cells (MOLM) were co-cultured with T-cells alone (upper illustration) or with T-cells together with autologous monocytes or AML-educated iMDSCs (n = 5) +/− AMG 330 (lower illustration). Specific lysis of MOLM-13 cells was assessed after 3 h. (**h**) Calcein-labeled monocytes or iMDSCs (*n* = 4) were co-cultured with autologous T-cells and MOLM-13 cells +/− AMG 330. Specific lysis of monocytes/iMDSCs was assessed after 3 h. Bars indicate the standard error of the mean. Abbreviations: *, *p* < 0.05; **, *p* < 0.01; ***, *p* < 0.001

In order to validate the AMG 330-triggered redirection of T-cells towards CD33^+^ AML-blasts AML-PBMC samples from newly diagnosed patients were used for short-term (three to six days) cell cultures in presence of control BiTE® constructs or AMG 330. In line with previous reports [8, 9], AMG 330 treatment resulted in an efficient elimination of CD33^+^ AML-blasts and the concomitant expansion of residual autologous T-cells (Fig. 2a). Most parameters that are indicative for T-cell activation (e.g. CD25 and CD69), cytotoxic activity (e.g. granzyme B and CD107), or cytokine production (e.g. IL2 and IFNγ) as well as the bystander activation of NK-cells (by amongst others abundant pro-inflammatory cytokines) were found upregulated upon AMG 330 application when phenotypically analyzing T- and NK-cells within the AML-PBMCs by FACS (Fig. 2b). The initial T-cell frequency within the PBMCs ranging from 0.16 to 14.30% and/or the initial MDSC levels had both no impact on the assessed levels of T-cell responsiveness (Additional file 1: Tables S1 and S2).

We next hypothesized that CD33^+^IDO^+^ MDSCs might antagonize AMG 330 efficacy by (A) competing over the target antigen CD33 or by (B) suppressing successfully recruited T-cells (by IDO). Using a FACS-based indirect QIFIKIT® immunofluorescence assay we quantified the CD33 cell surface antigen density (which is comparable for monocytes and AML-MDSCs, Fig. 1g) on primary AML-blasts and on CD14^+^ cells and indeed observed higher (potentially competing) CD33 levels on CD14^+^ cells (Fig. 2c). Concomitant blocking of the IDO activity using epacadostat in AML patient-derived PBMCs treated with AMG 330 resulted in a significantly enhanced reduction of the CD33^+^ cell count (Fig. 2d). Depleting all CD14^+^ cells (including monocytic MDSCs) in AML-PBMCs prior to AMG 330 treatment did not have a detectable effect on T-cell activation or their production of cytokines as well as T-cell expansion (Fig. 2e), which has been shown to be highly relevant for the clinical activity of BiTE® antibody constructs [18]. However, removal of all CD14^+^ cells led to an increased AMG 330-mediated lysis (Fig. 2f). For further investigating this phenomenon (in terms of being total CD14^+^ cell- or rather MDSC-driven), we cultured MOLM13 cells (cAML) with HD-derived T-cells and added AMG 330 in the presence of autologous HD-derived CD33^+^ monocytes or CD33^+^ iMDSCs. Co-cultures were performed for three days for preventing a reprogramming of the conventional monocytes into MDSCs that was occurring at day five (Fig. 1c and d). We observed a reduced specific lysis of MOLM13 cells only in co-cultures with iMDSCs (Fig. 2g) suggesting (at least ex vivo) a specifically MDSC-mediated (presumably only transient until MDSCs are eliminated by the redirected T-cells) reduction of AMG 330 efficacy most likely due to the MDSCs’ direct T-cell suppressive activity and not due to competition over the target antigen CD33 (which is found on both conventional monocytes and iMDSCs). At the same time BiTE-triggered lysis of iMDSCs and monocytes as shown in Fig. 1i remained unaffected in presence of MOLM13 AML-cells (Fig. 2h).

Taken together, our preclinical data suggests that AMG 330 could achieve anti-leukemic activity not only through direct engagement of T-cells but also via targeting of CD33^+^ monocytic MDSCs [1]. In accordance with our findings, recent preliminary data indicate that elimination of MDSCs by the bispecific CD33/CD3 T-cell engager AMV564 also restores immune homeostasis in MDS [19]. Furthermore, it remains to be elucidated whether MDSCs impact in vivo (at least temporarily until their AMG 330-triggered elimination) AMG 330 efficacy. In the latter case, MDSC levels could represent a biomarker for the patients’ clinical responsiveness towards an AMG 330-based therapy in analogy to observations from other immunotherapies such as peptide vaccination in renal cancer [20].

## Additional file

Additional file 1: **Table S1.** Fluorochrome-coupled antibodies and/or chemical dyes for flow cytometry. **Table S2.** AML-derived PBMCs (*n*=12) were treated with AMG 330 for three days. The median fluorescence intensity (MFI) of granzyme B (Grz B), CD107, perforin, CD69, CD137, CD25, CD154, IL2, IFNγ, and the cells’ expansion index was assessed by FACS in CD4^+^/CD8^+^ CD3^+^ T-cells as indicated. The association between those variables and the PBMCs’ initial frequency of HLA-DR^lo^ cells among CD14^+^ cells was calculated using a Pearson correlation analysis. Abbreviations: p, p-value; r, Pearson correlation. **Table S3.** AML-derived PBMCs (n=12) were treated with AMG 330 for three days. The median fluorescence intensity (MFI) of granzyme B (Grz B), CD107, perforin, CD69, CD137, CD25, CD154, IL2, and IFNγ was assessed by FACS in CD4^+^/CD8^+^ CD3^+^ T-cells. The association between those variables and the PBMCs’ initial frequency of CD3^+^ T-cells was calculated using a Pearson correlation analysis. Abbreviations: p, *p*-value; r, Pearson correlation. (DOCX 50 kb)

## Abbreviations

AML: Acute myeloid leukemia
CD: Cluster of differentiation
CLL: Chronic lymphocytic leukemia
FACS: Flow cytometry
IDO: Indoleamine-2,3-dioxygenase
MDS: Myelodysplastic syndrome
MDSC: Myeloid derived suppressor cell
PBMCs: Peripheral blood mononuclear cells
