# Supplementary material for: CD33/CD3-bispecific T-cell engaging (BiTE®) antibody construct targets monocytic AML myeloid-derived suppressor cells
Source: J Immunother Cancer. 2018 Nov 5;6:116. doi: 10.1186/s40425-018-0432-9 (PMC6217777; doi:10.1186/s40425-018-0432-9)
Supplement: Supplementary file 1 — Table S1. Fluorochrome-coupled antibodies and/or chemical dyes for flow cytometry. Table S2. AML-derived PBMCs (n=12) were treated with AMG 330 for three days. The median fluorescence intensity (MFI) of granzyme B (Grz B), CD107, perforin, CD69, CD137, CD25, CD154, IL2, IFNγ, and the cells’ expansion index was assessed by FACS in CD4+/CD8+ CD3+ T-cells as indicated. The association between those variables and the PBMCs’ initial frequency of HLA-DRlo cells among CD14+ cells was calculated using a Pearson correlation analysis. Abbreviations: p, p-value; r, Pearson correlation. Table S3. AML-derived PBMCs (n=12) were treated with AMG 330 for three days. The median fluorescence intensity (MFI) of granzyme B (Grz B), CD107, perforin, CD69, CD137, CD25, CD154, IL2, and IFNγ was assessed by FACS in CD4+/CD8+ CD3+ T-cells. The association between those variables and the PBMCs’ initial frequency of CD3+ T-cells was calculated using a Pearson correlation analysis. Abbreviations: p, p-value; r, Pearson correlation. (DOCX 50 kb) [file 40425_2018_432_MOESM1_ESM.docx]

**Additional file 1**

**Materials and methods**

**Patient material**

Samples were collected upon approval by the local ethics committee (number: 3779/138_17B) and patients’ informed consent. PBMCs from 13 newly diagnosed, untreated AML patients were collected.

**Antibodies, flow cytometry and FACS sorting**

Cells were stained according to the manufacturer`s recommendations using fluorochrome-coupled antibodies (Table S1). Cells were analyzed using a FACS Canto II cytometer (BD Biosciences, Franklin Lakes, NJ) and the FlowJo Version 10.1 software (TreeStar, Ashland, OR).

Staining was performed in presence of Fc-receptor blocking solution (Biolegend, San Diego, CA). Dead cells were excluded using the Zombie Aqua Fixable viability cell death marker (Biolegend). For intracellular cytokine staining cells were treated with Golgi Plug and the cytofix/cytoperm reagent (BD Bioscience). Surface density of CD33 was analyzed using the Agilent/Dako QIFIkit (Agilent Technologies, Santa Clara, CA).

| **Antibody** | **Fluorochrome** | **Clone** | **Isotype** | **Company** |
| --- | --- | --- | --- | --- |
| CD107a | APC Cy7 | H4A3 | Mouse IgG1, κ | Biolegend |
| CD117 | BV421 | 104 D2 | Mouse IgG1, κ | Biolegend |
| CD11b | APC | ICRF44 | Mouse IgG1, k | Biolegend |
| CD137 | PE Cy7 | 4B4-1 | Mouse IgG1, κ | Biolegend |
| CD14 | PerCP Cy5.5 | HCD 14 | Mouse IgG1, k | Biolegend |
| CD154 | FITC | 24-31 | Mouse IgG1, κ | Biolegend |
| CD16 | FITC | 3G8 | Mouse IgG1, κ | Biolegend |
| CD25 | PE | M-A251 | Mouse IgG1, κ | BD Bioscience |
| CD3 | APC | UCHT1 | Mouse IgG1, κ | Biolegend |
| CD3 | PerCP Cy5.5 | OKT 03 | Mouse IgG2a, κ | Biolegend |
| CD33 | APC Cy7 | P67.6 | Mouse IgG1, k | Biolegend |
| CD34 | PE Cy7 | 581 | Mouse IgG1, κ | Biolegend |
| CD4 | V500 | RPA-T4 | Mouse IgG1, κ | BD Bioscience |
| CD4 | FITC | OKT 04 | Mouse IgG2b, κ | Biolegend |
| CD45 | APC | HI30 | Mouse IgG1, κ | Biolegend |
| CD45 | V500 | HI30 | Mouse IgG1, k | BD Bioscience |
| CD56 | PE | HCD56 | Mouse IgG1, κ | Biolegend |
| CD69 | PerCP Cy5.5 | FN50 | Mouse IgG1, κ | Biolegend |
| CD8 | PerCP Cy5.5 | SK1 | Mouse IgG1, κ | Biolegend |
| CD8a | APC Cy7 | HIT8a | Mouse IgG1, κ | Biolegend |
| CPD670 | APC |  |  | eBioscience |
| Granzyme B | PB | GB11 | Mouse IgG1, κ | Biolegend |
| HLA-DR | FITC | G46-6 | IgG2a, k | BD Bioscience |
| IDO | PE | eyedio | Mouse IgG1, k | eBioscience |
| IFNγ | APC Cy7 | 4S.B3 | Mouse IgG1, κ | Biolegend |
| IL2 | PE | MQ1-17H12 | Rat IgG2a | Biolegend |
| Perforin | APC/PE-Cy7 | B-D48 | Mouse IgG1 | Biolegend |
| TNFα | APC | Mab11 | Mouse IgG1, κ | Biolegend |

Table S1: Fluorochrome-coupled antibodies and/or chemical dyes for flow cytometry.

**Cell preparation**

PBMCs were obtained using Ficoll-Paque (GE Healthcare, Piscateway Township, NJ). T-cells and monocytes were purified by magnetic bead-based negative selection (Pan T-cell and Pan Monocyte Isolation Kit, Miltenyi Biotec, Bergisch Gladbach, Germany). HLA-DR^lo^ and HLA-DR^hi^ CD14^+^ monocytes were purified by FACS-based sorting (FACS Aria II, BD Biosciences). Human AML cell-lines OCI-AML3, MOLM-13, and HL-60 were purchased from DSZM (Braunschweig, Germany).

**Cell culture**

AML-PBMCs were cultured at a density of 1.5x10^6^/ml in AIM-V® serum free medium (Thermo Fisher Scientific, Waltham, MA) at 37°C and 5% CO2 atmosphere in UpCell Plates (Nunc, Roskilde, Denmark). In indicated experiments 10 pM or 100 pM of AMG 330/control BITE^®^ (Amgen, Thousand Oaks, CA) was added as well as 1 µM epacadostat (Selleckchem, Munich, Germany). After three days T- and NK-cell activation was evaluated *via* FACS. After 6 days cell numbers were calculated utilizing BD Trucount Tubes (BD Bioscience) following the manufacturer’s instructions.

Monocytes were purified using a magnetic bead-based selection (Pan Monocyte Isolation Kit, Miltenyi Biotec). Next, monocytes were labeled with the cell dye eFluor® (Thermo Fisher Scientific, Waltham, MA) and subsequently co-cultured in UpCell Plates (Nunc) with AML blasts (in a 1 to 2.5 ratio) or with AML cell-lines (in a 4 to 1 ratio). Monocytes/MDSCs were separated by magnetic bead-based selection according to the phenotype (CD15, CD11b, and/or CD14 microbeads, Miltenyi Biotec).

**T-cell proliferation assay**

Monocytes/MDSCs and autologous VPD450-labeled CD3^+^ T-cells were cultured together and T-cells stimulated using anti-CD2, -CD3, and -CD28 activating microbeads (Miltenyi Biotec) as previously described^1^.

**T-cell activation and *ex vivo* expansion**

T-cells were expanded and pre-activated following a modified previously published protocol^2^. Expansion was performed for 9 days and T-cell aliquots were stored in liquid nitrogen. 24h prior use, cells were thawed and cultivated overnight in RPMI 10% FCS (Thermo Fischer Scientific).

**BITE^®^-dependent cellular cytotoxicity**

Target cells were labeled with calcein: monocytes with Calcein green and/or AML-cells with Calcein red-orange AM (Invitrogen, Carlsbad, CA). Specific lysis of target cells based on the released calcein was calculated as previously detailed^2^. Target cells (monocytes, MDSCs, and AML-cells or monocytes/MDSCs with AML-cells) were co-cultured with pre-stimulated T-cells in presence or absence of AMG 330/control BITE^®^ in a 1 to 10 ratio for 3h. Calcein release was measured at 485/525nm for Calcein green and at 350/589nm for Calcein red-orange.

**Cell conjugation assays**

Myeloid cells stained with anti-CD14 antibodies were cultured with magnetic-bead isolated (Pan T cell isolation kit, Miltenyi Biotec) pre-activated T-cells at a ratio of 1 to 1 for 6 minutes. Next, cells were fixed with 1.5% formaldehyde for 30 minutes at room temperature. Cells were washed and T-cells stained with an anti-CD3 or anti-CD8 antibody. Permeabilization was performed in PBS containing 2% FCS and 0.1% Triton X. Actin was stained using Biotin-XX Phalloidin and Streptavidin PE Texas Red (Thermo Fisher Scientific). In selected experiments perforin pore delivery at the cytotoxic immune synapse was visualized by perforin staining. Nuclear counter staining was done with SYTO13 (Thermo Fisher Scientific). Cells were analyzed using an AMNIS flow sight FACS-imaging device (Merck Millipore, Darmstadt, Germany).

### RNA preparation and quantitative polymerase chain reaction (qPCR)

For quantitative polymerase chain reaction (qPCR) total RNA was extracted from cell lysates with innuPREP, RNA Mini Kit (Analytic Jena, Jena, Germany). Complementary DNA was prepared using a Superscript First Strand Synthesis System (Life Technologies, Carlsbad, CA) on a Mastercycler Nexus (Eppendorf, Hamburg, Germany). Messenger RNA levels were quantified by qPCR utilizing Quantitect SYBR Green (Qiagen, Hilden, Germany) on a Rotor Gene Q device (Qiagen). Relative gene expression was determined by normalizing the expression of each target gene to β*-actin* (Hs-ACTB_2_SG, Qiagen) using gene-specific primer (Hs_IDO1_1-SG, Qiagen).

**Statistical analyses**

Differences in means were evaluated using a paired/unpaired t-test if not stated otherwise. All statistical analyses were performed using GraphPad Prism Version 7 (GraphPad Prism Software Inc.).

**References**

^1^Jitschin R, Braun M, Buttner M, et al. CLL-cells induce IDO^hi^ CD14^+^HLA-DR^lo^ myeloid-derived suppressor cells that inhibit T-cell responses and promote T_Regs_. *Blood*. 2014;124(5):750-760.

^2^Schubert I, Saul D, Nowecki S, et al. A dual-targeting triplebody mediates preferential redirected lysis of antigen double-positive over single-positive leukemic cells. *MAbs*. 2014;6(1):286-96.

**Tables**

| **%HLA-DR^lo^ [among CD14+] *vs.*** | **AMG 330** | | | |
| --- | --- | --- | --- | --- |
|  | **10 pM** | | **100 pM** | |
|  | **r** | **p** | **r** | **p** |
| **CD154 MFI [CD4+]** | 0,20 | 0,53 | 0,24 | 0,46 |
| **CD25 MFI [CD4+]** | -0,09 | 0,79 | 0,00 | 1,00 |
| **CD137 MFI [CD4+]** | 0,26 | 0,42 | 0,33 | 0,30 |
| **CD69 MFI [CD4+]** | 0,14 | 0,67 | 0,14 | 0,68 |
| **IFNγ MFI [CD4+]** | -0,13 | 0,68 | -0,09 | 0,77 |
| **IL2 MFI [CD4+]** | 0,16 | 0,61 | 0,02 | 0,95 |
| **CD154 MFI [CD8+]** | 0,02 | 0,94 | 0,17 | 0,60 |
| **CD25 MFI [CD8+]** | -0,14 | 0,67 | 0,08 | 0,81 |
| **CD137 MFI [CD8+]** | 0,02 | 0,96 | 0,14 | 0,66 |
| **CD69 MFI [CD8+]** | -0,13 | 0,68 | -0,05 | 0,88 |
| **IFNγ MFI [CD8+]** | 0,11 | 0,74 | -0,05 | 0,88 |
| **IL2 MFI [CD8+]** | 0,05 | 0,88 | 0,04 | 0,91 |
| **perforin MFI [CD3+]** | 0,45 | 0,17 | 0,18 | 0,62 |
| **CD107 MFI [CD3+]** | 0,17 | 0,62 | -0,04 | 0,92 |
| **GrzB MFI [CD3+]** | 0,26 | 0,44 | 0,06 | 0,86 |
| **Expansion Index [CD3+]** | 0,29 | 0,37 | 0,19 | 0,56 |

**Table S2:** AML-derived PBMCs (n=12) were treated with AMG 330 for three days. The median fluorescence intensity (MFI) of granzyme B (Grz B), CD107, perforin, CD69, CD137, CD25, CD154, IL2, IFNγ, and the cells’ expansion index was assessed by FACS in CD4^+^/CD8^+^ CD3^+^ T-cells as indicated. The association between those variables and the PBMCs’ initial frequency of HLA-DR^lo^ cells among CD14^+^ cells was calculated using a Pearson correlation analysis. Abbreviations: p, p-value; r, Pearson correlation.

| **%CD3+ T-cells *vs.*** | **AMG 330** | | | |
| --- | --- | --- | --- | --- |
|  | **10 pM** | | **100 pM** | |
|  | **r** | **p** | **r** | **p** |
| **CD154 MFI [CD4+]** | -0,09 | 0,79 | -0,14 | 0,67 |
| **CD25 MFI [CD4+]** | 0,38 | 0,24 | 0,26 | 0,44 |
| **CD137 MFI [CD4+]** | 0,11 | 0,75 | -0,27 | 0,41 |
| **CD69 MFI [CD4+]** | -0,29 | 0,38 | -0,28 | 0,41 |
| **IFNγ MFI [CD4+]** | 0,14 | 0,69 | 0,13 | 0,71 |
| **IL2 MFI [CD4+]** | -0,34 | 0,30 | -0,24 | 0,47 |
| **CD154 MFI [CD8+]** | -0,40 | 0,22 |  |  |
| **CD25 MFI [CD8+]** | 0,09 | 0,79 |  |  |
| **CD137 MFI [CD8+]** | -0,21 | 0,54 |  |  |
| **CD69 MFI [CD8+]** | -0,10 | 0,77 |  |  |
| **IFNγ MFI [CD8+]** | -0,29 | 0,38 | -0,29 | 0,39 |
| **IL2 MFI [CD8+]** | -0,28 | 0,40 | -0,40 | 0,22 |
| **perforin MFI [CD3+]** | -0,45 | 0,20 |  |  |
| **CD107 MFI [CD3+]** | -0,11 | 0,77 |  |  |
| **GrzB MFI [CD3+]** | -0,25 | 0,49 |  |  |

**Table S3:** AML-derived PBMCs (n=12) were treated with AMG 330 for three days. The median fluorescence intensity (MFI) of granzyme B (Grz B), CD107, perforin, CD69, CD137, CD25, CD154, IL2, and IFNγ was assessed by FACS in CD4^+^/CD8^+^ CD3^+^ T-cells. The association between those variables and the PBMCs’ initial frequency of CD3^+^ T-cells was calculated using a Pearson correlation analysis. Abbreviations: p, p-value; r, Pearson correlation.
